# Colonic leiomyoma mimicking a liver tumor: an unusual diagnosis after en-block robotic resection

**DOI:** 10.1093/jscr/rjab418

**Published:** 2021-09-22

**Authors:** Nicolás H Dreifuss, Alberto Mangano, Gabriela Aguiluz, Vikas Mehta, Sean Koppe, Charles Berkelhammer, Pier C Giulianotti

**Affiliations:** Division of General, Minimally Invasive, and Robotic Surgery, Department of Surgery, University of Illinois at Chicago, Chicago, IL, USA; Division of General, Minimally Invasive, and Robotic Surgery, Department of Surgery, University of Illinois at Chicago, Chicago, IL, USA; Division of General, Minimally Invasive, and Robotic Surgery, Department of Surgery, University of Illinois at Chicago, Chicago, IL, USA; Department of Pathology, University of Illinois at Chicago, Chicago, IL, USA; Division of Gastroenterology and Hepatology, Department of Medicine, University of Illinois at Chicago, Chicago, IL, USA; Department of Gastroenterology, Advocate-Aurora Christ Medicine Center, Oak Lawn, IL, USA; Division of General, Minimally Invasive, and Robotic Surgery, Department of Surgery, University of Illinois at Chicago, Chicago, IL, USA

## Abstract

Colonic leiomyomas are rare. Their clinical presentation ranges from asymptomatic polyps detected on endoscopy to large symptomatic abdominopelvic masses. Imaging findings are usually non-specific, and percutaneous biopsy might help with differential diagnosis. However, radical surgery with negative margins is ultimately needed to rule out malignancy. We describe an uncommon presentation of a colonic leiomyoma mimicking a right hepatic lobe tumor on preoperative imaging. The robotic approach allowed a precise abdominal exploration with confirmation of colonic and hepatic infiltration and subsequent oncological en-block resection. Surgeons operating on hepatic tumors close to the right colic flexure should be aware of this diagnosis.

## INTRODUCTION

Leiomyomas of the alimentary tract are usually located in the esophagus, stomach, or the small intestine and more rarely in the colon [1]. Some patients present with mechanical obstruction, bleeding, abdominal pain or palpable abdominal mass [2]. Colonic leiomyomas arising from the muscularis propria tend to grow extramurally, producing large masses that remain asymptomatic for a long time. Imaging findings are generally unspecific and preoperative biopsy might orientate the diagnosis. However, surgical resection is required to confirm the diagnosis and rule out malignancy [3–4]. Here, we describe an unusual presentation of a colonic leiomyoma presenting as a right lobe liver mass on preoperative imaging.

## CASE REPORT

**Figure 1:**
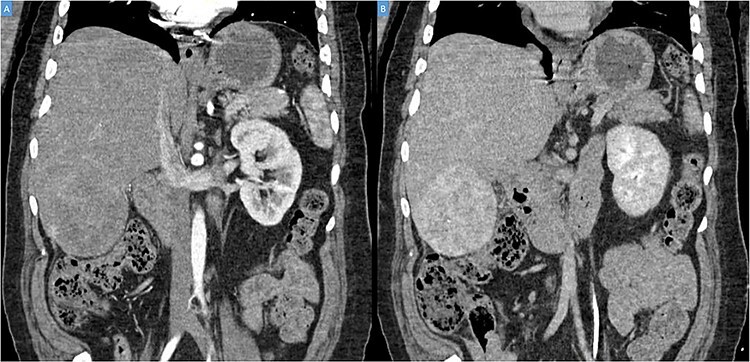
(**A**, **B**) Coronal computed tomography of the abdomen showing a large tumor on liver’s segments V–VI with heterogenous contrast enhancement.

**Figure 2:**
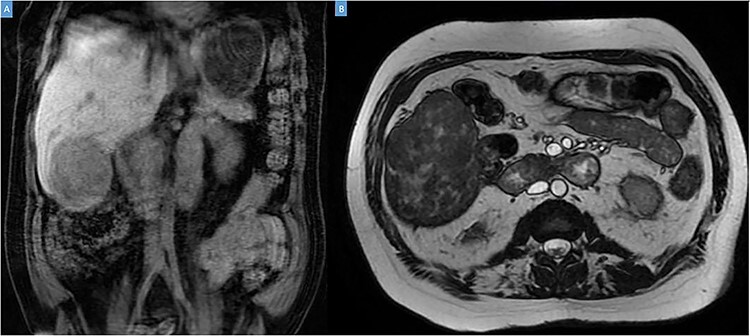
Coronal (**A**) and axial (**B**) sections of MRI showing the peripheral liver tumor.

A 49-year-old male presented with a large right hepatic lobe mass on abdominal computed tomography (CT) scan ordered for non-specific abdominal pain. He had a past medical history of type 2 diabetes mellitus, glaucoma and deafness due to mumps. The physical examination was normal, with a body mass index of 22 kg/m^2^ and no previous abdominal surgeries. CT scan (Fig. 1A and B) and magnetic resonance imaging (MRI; Fig. 2A and B) revealed a 10.7 × 7.6 × 8.9-cm mass involving the liver’s segments V–VI with heterogeneous enhancement and areas of calcification.

An ultrasound-guided liver biopsy indicated a spindle cell neoplasm with smooth muscle differentiation. Liver function tests were within normal limits. The case was extensively discussed by a multidisciplinary team, and there was consensus to proceed to surgery with the diagnosis of resectable peripheral liver tumor.

**Figure 3:**
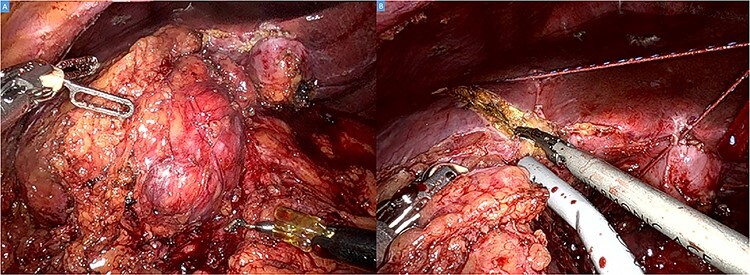
Intraoperative picture showing a bulky tumor infiltrating the liver’s segments V–VI and the right colic flexure (**A**). Hepatic transection with the harmonic (**B**).

The operation was performed using the Da Vinci Xi system. Initial exploration showed a large and bulky lesion in the liver’s segments V–VI completely fused to the right colonic flexure (Fig. 3A and B).

The ascending colon and hepatic flexure were detached. The mesocolon was divided, and the ascending and proximal transverse colon were transected with the stapler leaving the right hepatic flexure attached to the tumor. Before the hepatic transection, intraoperative ultrasound was performed to confirm the tumor position, plan the resection line and rule out other nodules. Intestinal continuity restoration was achieved with a side-to-side anastomosis. Finally, the specimen was retrieved through a Pfannenstiel incision.

**Figure 4:**
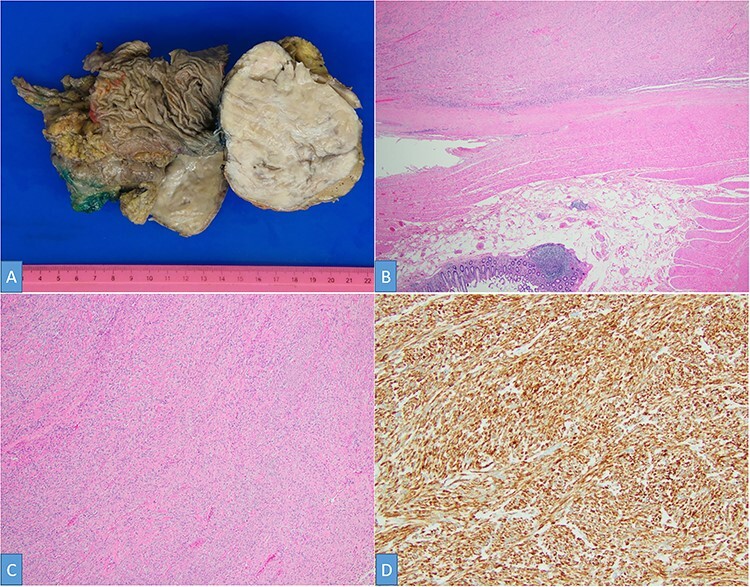
Gross examination shows a solid white-tan tumor arising from colonic wall and pushing into the liver parenchyma (**A**). A cellular spindle cell neoplasm can be seen arising from colonic muscular wall; H&E ×20 (**B**). The tumor showed no increase in cellularity, mitotic figures or foci of necrosis; H&E ×100 (**C**). SMA immunohistochemistry ×200 (**D**).

The postoperative course was uneventful, and the patient was discharged on postoperative Day 5. At 2 weeks postoperatively, the patient presented with fever, and a CT scan demonstrated an infected subhepatic collection. The patient was readmitted, and the collection was successfully treated with antibiotics and percutaneous drainage. The surgical specimen showed a hard, round 10.8 × 8.9 × 7.2-cm mass bulging out from the liver’s surface and adhered to the right colic flexure (Fig. 4A). Histologically, the tumor was composed of brightly eosinophilic spindled cells with blunt-ended nuclei and low cellularity (Fig. 4B). No mitotic figure or foci of necrosis, as well as significant nuclear atypia, are identified (Fig. 4C). The tumor cells are positive for smooth muscle actin (SMA), desmin, CD117 (focal) and are negative for DOG1 and CD34 (Fig. 4D). These findings support a diagnosis of colonic leiomyoma. Liver parenchyma and large bowel margins were negative.

## DISCUSSION

Smooth muscle tumors are the second most common mural mesenchymal neoplasia of the gastrointestinal tract after gastrointestinal stromal tumors (GIST; [5]). Colonic leiomyomas are rare and represent 3–15% of all alimentary tract smooth muscle tumors [1, 5–7]. These tumors arise most commonly from the muscularis propria or mucosae of the intestinal wall. However, they can also take origin from media of the blood vessels and therefore occur in any organ or tissue. Usually, they are unifocal but may be multiple in the leiomyomatosis syndrome [8]. Clinical presentation varies from an asymptomatic polyp/mass (incidentally found on colonoscopy or imaging) to complications such as perforation leading to peritonitis, ulceration and bleeding, intussusception and bowel obstruction requiring urgent surgical management [3–4, 9, 10].

Imaging findings are usually non-specific, and consist of tumors with lobulated margins, areas of calcification, focal necrosis, cystic changes and heterogeneous enhancement with contrast. These findings can be seen both in benign and malignant conditions [11]. Despite some features may be more suggestive of malignancy (size >5 cm, mesenteric fat infiltration, ulceration, regional lymphadenopathy and exophytic grow pattern), surgical resection of the mass is ultimately necessary to exclude malignancy. Interestingly, our patient’s colonic leiomyoma was mimicking a right lobe liver tumor. Preoperative imaging suggested a peripheral hepatic mass but did not help to determine its colonic origin or infiltration. Radiologically, the lesion was not typical for any of the more common hepatic benign or malignant neoplasms. It seems that imaging alone cannot reliably differentiate between primary leiomyoma and other potential differential diagnoses. In our case, it was the percutaneous biopsy that confirmed the smooth muscle origin. Another potential differential diagnosis is a primary hepatic leiomyoma (PHL). PHL are rare tumors that may arise from the smooth muscle of the liver vessels or bile duct [12]. However, the diagnosis of PHL should meet the following diagnostic criteria: presence of leiomyocytes within the liver lesion and no evidence of a smooth muscle tumor elsewhere within the body [13].

Histologically, the most important differential diagnosis of leiomyoma is GIST and malignant leiomyosarcoma. The distinction between leiomyoma and leiomyosarcoma is challenging and is based on the presence of necrosis, tumor size, mitotic count, atypia and nuclear pleomorphism [5]. Moreover, immunohistochemistry analysis is useful to differentiate leiomyomas from GIST. Leiomyomas usually stain positive for SMA and desmin but negative to CD117 and CD34. Conversely, GIST reacts positive to CD34, CD117 and almost 30% to SMA [14]. Unlike leiomyosarcomas, the prognosis of colonic leiomyomas is good, and resection is usually curative [3].

Different techniques have been employed to treat these tumors. Small endoluminal leiomyomas could be endoscopically resected with excellent outcomes [3, 15]. However, surgical resection is necessary for most leiomyomas. Minimally invasive surgery for liver and colon resections has a defined role. Robotic resection of hepatic and colonic tumors has proved to be safe and feasible with at least comparable outcomes to both open and laparoscopic approaches [16–18]. Although the laparoscopic approach is widely accepted for colonic resections, more complex hepatic or multi-visceral resections are challenging [19]. In the present case, the robotic platform allowed a precise en-block oncological resection while maintaining all the benefits of the minimally invasive approach.

To the best of our knowledge, this is the first reported case of a colonic leiomyoma presenting as a hepatic mass. Neoplasms involving the right colic flexure should be considered in the differential diagnosis of segments V–VI hepatic masses.

## ETHICS APPROVAL AND CONSENTS

This study was performed in accordance with the Declaration of Helsinki, the institutional review board (IRB) approval of our institution, and informed patient consent was obtained for case publication.

## AUTHORS' CONTRIBUTIONS

NHD, SK, CB and PCG: Conception and design. NHD, AM, GA and VM: Data acquisition, analysis and interpretation of data. NHD, AM, GA, VM, SK, CB and PCG: Drafting of manuscript, and critical revision of manuscript. All authors read and approved the final manuscript.

## Data Availability

Data sharing is not applicable to this article as no datasets were generated or analyzed during the current study.
